# Select estrogens within the complex formulation of conjugated equine estrogens (Premarin^®^) are protective against neurodegenerative insults: implications for a composition of estrogen therapy to promote neuronal function and prevent Alzheimer's disease

**DOI:** 10.1186/1471-2202-7-24

**Published:** 2006-03-13

**Authors:** Liqin Zhao, Roberta Diaz Brinton

**Affiliations:** 1 Department of Molecular Pharmacology and Toxicology, Norris Foundation Laboratory for Neuroscience Research, University of Southern California, Pharmaceutical Sciences Center, Los Angeles, CA 90089, USA

## Abstract

**Background:**

Results of the Women's Health Initiative Memory Study (WHIMS) raised concerns regarding the timing and formulation of hormone interventions. Conjugated equine estrogens (CEE), used as the estrogen therapy in the WHIMS trial, is a complex formulation containing multiple estrogens, including several not secreted by human ovaries, as well as other biologically active steroids. Although the full spectrum of estrogenic components present in CEE has not yet been resolved, 10 estrogens have been identified. In the present study, we sought to determine which estrogenic components, at concentrations commensurate with their plasma levels achieved following a single oral dose of 0.625 mg CEE (the dose used in the WHIMS trial) in women, are neuroprotective and whether combinations of those neuroprotective estrogens provide added benefit. Further, we sought, through computer-aided modeling analyses, to investigate the potential correlation of the molecular mechanisms that conferred estrogen neuroprotection with estrogen interactions with the estrogen receptor (ER).

**Results:**

Cultured basal forebrain neurons were exposed to either β-amyloid_25–35 _or excitotoxic glutamate with or without pretreatment with estrogens followed by neuroprotection analyses. Three indicators of neuroprotection that rely on different aspects of neuronal damage and viability, LDH release, intracellular ATP level and MTT formazan formation, were used to assess neuroprotective efficacy. Results of these analyses indicate that the estrogens, 17α-estradiol, 17β-estradiol, equilin, 17α-dihydroequilin, equilinen, 17α-dihydroequilenin, 17β-dihydroequilenin, and Δ^8,9^-dehydroestrone were each significantly neuroprotective in reducing neuronal plasma membrane damage induced by glutamate excitotoxicity. Of these estrogens, 17β-estradiol and Δ^8,9^-dehydroestrone were effective in protecting neurons against β-amyloid_25–35_-induced intracellular ATP decline. Coadministration of two out of three neuroprotective estrogens, 17β-estradiol, equilin and Δ^8,9^-dehydroestrone, exerted greater neuroprotective efficacy than individual estrogens. Computer-aided analyses to determine structure/function relationships between the estrogenic structures and their neuroprotective activity revealed that the predicted intermolecular interactions of estrogen analogues with ER correlate to their overall neuroprotective efficacy.

**Conclusion:**

The present study provides the first documentation of the neuroprotective profile of individual estrogens contained within the complex formulation of CEE at concentrations commensurate with their plasma levels achieved after an oral administration of 0.625 mg CEE in women. Our analyses demonstrate that *select *estrogens within the complex formulation of CEE contribute to its neuroprotective efficacy. Moreover, our data predict that the magnitude of neuroprotection induced by individual estrogens at relatively low concentrations may be clinically undetectable and ineffective, whereas, a combination of select neuroprotective estrogens could provide an increased and clinically meaningful efficacy. More importantly, these data suggest a strategy for determining neurological efficacy and rational design and development of a composition of estrogen therapy to alleviate climacteric symptoms, promote neurological health, and prevent age-related neurodegeneration, such as AD, in postmenopausal women.

## Background

Multiple factors have been hypothesized as potential contributors to the disparity between observational studies, which found that estrogen/hormone therapy (ET/HT) is associated with improved cognitive function and/or reduced risk (20–50%) of developing Alzheimer's disease (AD) in postmenopausal women [1-6], and the randomized double blind clinical trial, Women's Health Initiative Memory Study [7-10], or trials of ET/HT in women with existing AD [11-14], where ET/HT showed no benefit and in some instances adverse outcomes on neurological health in postmenopausal women. Prime among those factors are the temporal parameters of ET/HT intervention and the ET/HT formulation.

Using a prevention model paradigm in which cultured primary neurons were treated with conjugated equine estrogens (CEE) prior to exposure to degenerative insults associated with AD, our earlier work demonstrated that CEE significantly protected neurons against toxic insults-induced cell death [15]. In a treatment model paradigm, in which primary neurons were first exposed to β-amyloid_25–35 _followed by CEE treatment, neurons treated with CEE had a slower rate of degeneration but were not protected against β-amyloid_25–35_-induced cell death [15]. Our *in vitro *model systems that simulate prevention versus treatment modes of estrogen exposure indicate a healthy cell bias of estrogen action in neurons [16,17], and they provide us a lens through which to view the clinical data from both observational studies and trials. That is, women who received ET/HT at the time of menopause, in a prevention mode before extensive age-associated degeneration occurs, exhibited improved cognitive function and a lower risk of developing AD than women who had never received ET/HT, which is consistent with most observational analyses [1-6,18-21]. In contrast, when women received ET/HT in their 60's or 70's, when age-associated insults have already occurred in some women, or following the onset of AD, ET/HT had no benefit to or even exacerbated the degenerative state, as demonstrated in a number of clinical trials, including the WHIMS [7-14]. These analyses suggest that ET/HT can be effective in preventing rather than treating age-related neuronal decline and degeneration.

Timing of ET/HT intervention is paralleled by the crucial issue of ET/HT formulation. First, the question of which progestin to use in ET/HT remains unresolved. Results of the WHIMS CEE plus medroxyprogesterone acetate (MPA) trial indicated an increased risk (hazard ratio: 2.05) of developing dementia [8,10], whereas the CEE alone trial showed no statistically significant benefit or detriment, although there was a trend towards increased risk (hazard ratio: 1.49) of developing dementia [7,9]. The difference in severity of adverse outcomes between the CEE plus MPA trial and CEE alone trial suggests that the addition of MPA to the estrogen formulation exacerbated neurological decline in postmenopausal women aged 65 years or older. These clinical findings are consistent with our *in vitro *data demonstrating that MPA antagonized 17β-estradiol-induced mechanisms of memory and neuroprotection. In contrast, progesterone was both neuroprotective alone and synergized with 17β-estradiol [22-25].

**Figure 1:**
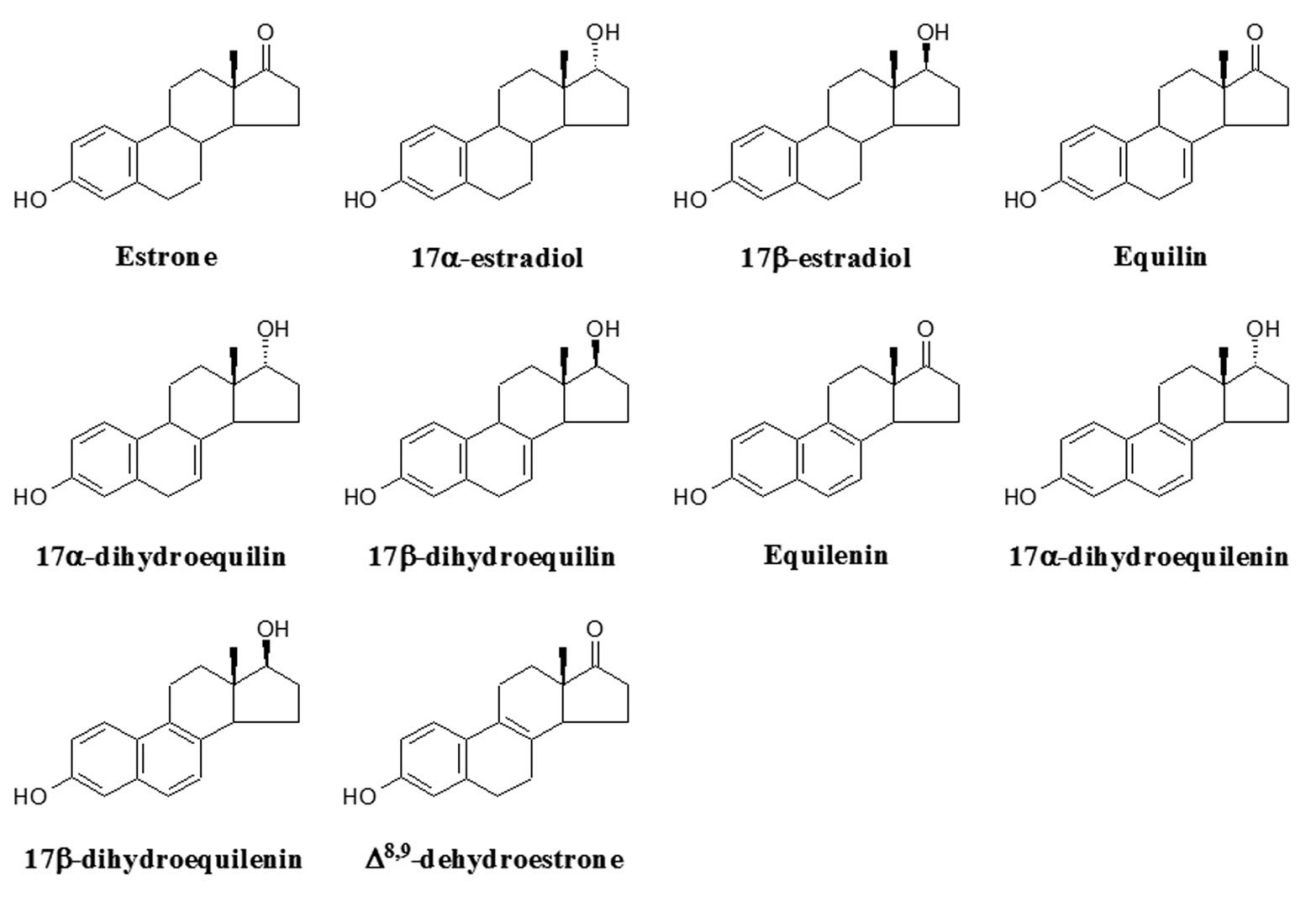
Chemical structures of test estrogens within CEE.

Moreover, the estrogen component of ET/HT can affect study outcomes as well. Earlier trials have revealed a trend of better cognitive scores from a single estrogen formulation, 17β-estradiol valerate [26-28], than CEE, a complex formulation of estrogens [29,30]. Although CEE showed beneficial effects in some prevention model systems, this complex formulation, extracted from pregnant mares' urine, contains multiple estrogens, including ones not secreted by human ovaries, as well as other biologically active steroids [31]. Whether this complex formulation of ET/HT, as well as the doses commonly used in clinical studies, are optimal to provide the maximum neurological benefit remains to be determined. Although the full spectrum of estrogenic components present in CEE has not yet been resolved, 10 estrogens have been identified (Fig. 1), including the sulfate esters of the ring B saturated estrogens (classical estrogens), estrone, 17α-estradiol and 17β-estradiol, and the ring B unsaturated estrogens, equilin, 17α-dihydroequilin, 17β-dihydroequilin, equilenin, 17α-dihydroequilenin, 17β-dihydroequilenin and Δ^8,9^-dehydroestrone [31]. Studies have indicated that all these estrogens bind to estrogen receptor (ER), though with different affinities, and are uterotrophic as demonstrated by increased uterine weight in rats [31,32].

The current study was undertaken to determine the impact of the above 10 estrogenic components within CEE on measures of neuronal viability and survival, at concentrations commensurate with their plasma levels achieved following a single oral dose of 0.625 mg CEE [33]. Subsequently, combinations of neuroprotective estrogens were assessed to determine whether coadministration of select estrogenic molecules within CEE were more efficacious than single molecule formulations. Primary cultures of basal forebrain neurons were used due to their vulnerability to neurodegeneration in the aging or AD brain [34,35], and all analyses were conducted with a comparative reference to 17β-estradiol. Further, computer-aided modeling analyses were conducted to investigate the potential correlation among the neuroprotective efficacy of estrogens, their structures and estrogen interactions with ER.

## Results

### Select estrogens within CEE are neuroprotective against β-amyloid_25–35_-induced toxicity in primary basal forebrain neurons

Our earlier work demonstrated that the complex formulation of CEE is significantly protective against β-amyloid_25–35_-induced neurotoxicity in primary basal forebrain neurons [15]. To determine which of the estrogenic components within CEE exert neuroprotective effects, cultured basal forebrain neurons were pretreated with test estrogens for 4 days at concentrations commensurate with those detected in plasma following a single oral dose of 0.625 mg CEE followed by exposure to a toxic level of β-amyloid_25–35 _(8 μg/ml) for 24 hr. As shown in Table 1, select estrogens contained within CEE were neuroprotective against β-amyloid_25–35_-induced toxic insult. At concentrations comparable to plasma levels of estrogens, 17α-estradiol, 17β-estradiol, equilin, equilenin, 17α-dihydroequilin and Δ^8,9^-dehydroestrone, each significantly reduced the β-amyloid_25–35_-induced LDH release. The LDH level from cultures pretreated with estrone or 17β-dihydroequilin was not significantly different compared to β-amyloid_25–35 _alone-treated cultures (Table 1).

### Differential neuroprotective profile with intracellular ATP level as the neuronal viability index

Pretreatment with 17β-estradiol, estrone and Δ^8,9^-dehydroestrone, individually, significantly protected neurons against β-amyloid_25–35_-induced intracellular ATP decline (Table 2). Neurons pretreated with equilin had a nonsignificant increase in intracellular ATP level compared to β-amyloid_25–35 _alone-treated cultures (Table 2). In contrast, 17α-estradiol, equilenin and 17α-dihydroequilin, each of which was demonstrated to be neuroprotective by LDH measurements, were ineffective in protecting basal forebrain neurons against β-amyloid_25–35_-induced ATP decline (Table 2).

### Neuroprotective effects of select estrogens against glutamate-induced excitotoxicity

We previously demonstrated that 17β-estradiol, equilin and Δ^8,9^-dehydroestrone are neurotrophic in promoting neurite outgrowth [36,37]. Results of the present study further indicated that these 3 estrogens were neuroprotective against LDH release and intracellular ATP decline following compromised neuronal plasma membrane integrity and mitochondrial function induced by β-amyloid_25–35_. We then tested these estrogens for their neuroprotective efficacy against excitotoxic insult by 200 μM glutamate. Estrone was examined as well based on its significant effect in preventing the intracellular ATP decline induced by β-amyloid_25–35_. Data shown in Table 3 demonstrate that the estrogens, 17β-estradiol, equilin and Δ^8,9^-dehydroestrone, but not estrone, significantly protected neurons against neurotoxic glutamate-induced LDH release. Further analyses of neuronal viability of intracellular ATP content revealed that none of these estrogens significantly protected neurons against excitotoxic glutamate-induced ATP decline (Table 4).

### Neuroprotective effects of select estrogens based on MTT reduction measurements

To further determine the impact of select estrogens on neuronal metabolic capability, the mitochondrial conversion of MTT to formazan crystals by metabolically active neurons was measured in cultures pretreated with estrogens followed by exposure to excitotoxic glutamate. Select neuroprotective estrogens determined by LDH measurements as shown in Table 1, as well as two other estrogens, 17α-dihydroequilenin and 17β-dihydroequilenin, that were not tested previously, were evaluated. Both LDH and MTT assay were conducted on cultures from the same plate (Table 5). Results from the LDH measurements were consistent with the previous data, indicating that estrogens, 17α-estradiol, 17β-estradiol, equilin, equilenin, 17α-dihydroequilin, and Δ^8,9^-dehydroestrone were neuroprotective against neuronal membrane damage induced by exposure to both β-amyloid_25–35 _and glutamate (Table 1 and 5). Both 17α-dihydroequilenin and 17β-dihydroequilenin significantly reduced the glutamate-induced LDH release as well. Consistent with the results from ATP analysis, none of the estrogens at the test concentrations protected neurons against the glutamate-induced decline of metabolic activity in basal forebrain neurons.

### Coadministration of select neuroprotective estrogens exerts greater efficacy against neurotoxic insult

**Figure 2:**
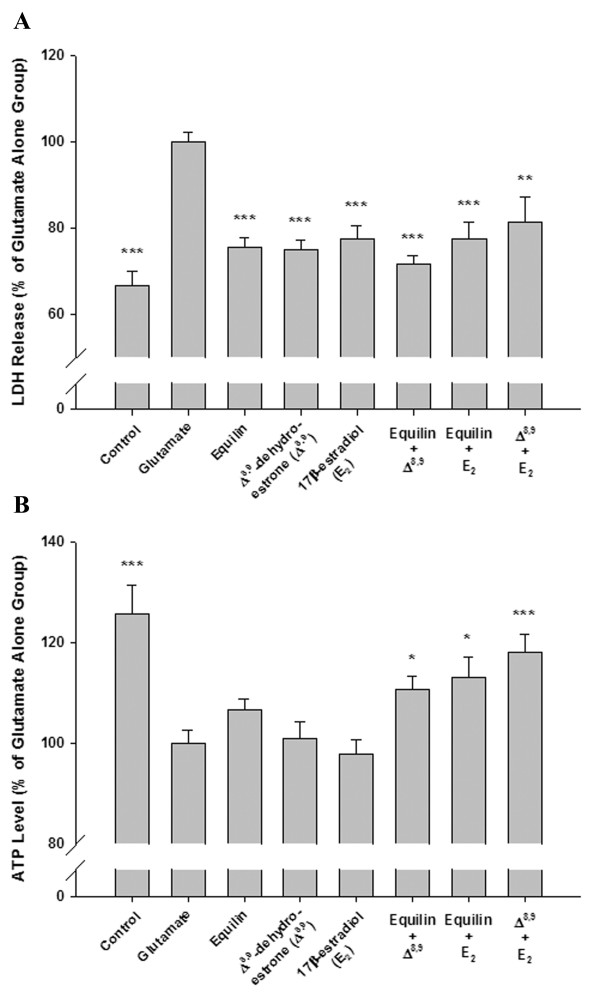
**Efficacy of combined administration of select neuroprotective estrogens within CEE against glutamate-induced toxicity in basal forebrain neurons**. Three-day-old basal forebrain neurons were pretreated with either single estrogens or combinations of estrogens followed by a 5 min exposure to 200 μM glutamate. Both LDH release into the medium and the intracellular ATP level were measured 24 hr later. Data are represented as mean ± SEM (n = 8 – 12 from 3 experiments). * *P *< 0.05, ** *P *< 0.01 and *** *P *< 0.001 compared to glutamate alone-treated cultures.

17β-Estradiol, equilin and Δ^8,9^-dehydroestrone, which were effective in protecting basal forebrain neurons against both β-amyloid_25–35 _and glutamate-induced damage to membrane integrity, as well as β-amyloid_25–35_-induced decline in mitochondrial function, were further evaluated for their neuroprotective efficacy when coadministered in cultured neurons. Results shown in Fig. 2A demonstrate that the LDH release from cultures pretreated with combinations of two out of these three estrogens were not significantly different from cultures pretreated with single estrogens. However, all three combinations significantly protected neurons against intracellular ATP decline compared to either glutamate alone-treated cultures or cultures pretreated with single estrogens (Fig. 2B). These data indicate that greater neuroprotective efficacy could be achieved by the coadministration of those neuroprotective estrogens at the concentrations consistent with their plasma levels achieved following an oral administration of 0.625 mg of CEE.

### Correlation between the interactions with ER and the neuroprotective profile of estrogen analogues

**Figure 3:**
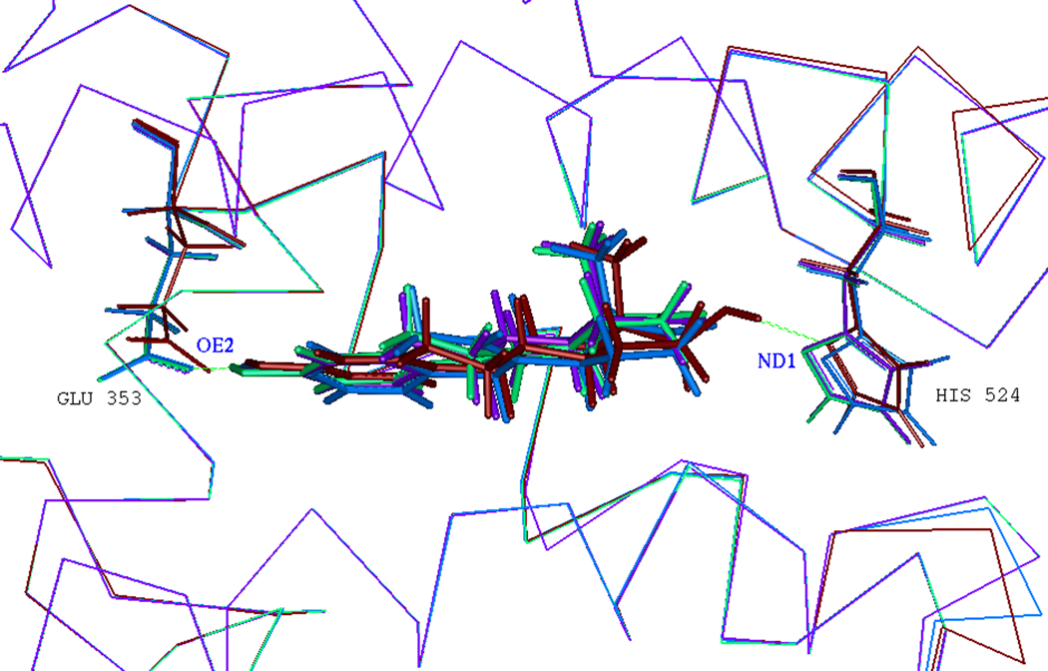
**Superimposition of 3D complex models of ERα with 17β-estradiol (dark red; PDB ID: 1ERE), equilin (blue), Δ^8,9^-dehydroestrone (green) and estrone (purple)**. Estrogen receptors are represented as alpha carbon traces. Hydrogen bonds are labeled in dashed green lines. The comparison of these complex structures revealed that the four estrogens bind to ERα in a similar binding mode, with a hydrogen bond interaction occurring between the atom OE2 of the residue Glu 353 in the binding site of the receptor and the hydrogen atom of the phenol group in the A ring of estrogens. An additional hydrogen bond interaction occurs between the atom ND1 of the residue His 524 and the hydroxyl group in the D ring of 17β-estradiol. Image was generated using Accelrys InsightII 2000.

To gain insights into the intrinsic relationship between the estrogen structures and their neuroprotective activities, we computationally simulated the interactions of 4 representative estrogens with the ligand binding site of ER. In light of our previous finding of equivalent contribution of ER subtypes, α and β, to estrogen neuroprotection, we used ERα as the docking target in this analysis. Three estrogens, 17β-estradiol, equilin and Δ^8,9^-dehydroestrone, consistently and significantly protected basal forebrain neurons against neurotoxic insults, while estrone was ineffective in most measurements. Results of molecular docking revealed that all 4 estrogens bind to ERα in a similar binding mode, with a hydrogen bond interaction occurring in all complexes between the atom OE2 of the residue Glu 353 in the binding site of the receptor and the hydrogen atom of the phenol group in the A ring of estrogens (Fig. 3). Rank order of the intermolecular energy, a total of both the van der Waals (VDW) and electrostatic energy between estrogens and ERα, indicated that 17β-estradiol > equilin and Δ^8,9^-dehydroestrone > estrone (Table 6), which is parallel to their neuroprotective activities. In addition to the hydrogen bonding derived from the A ring phenol group which characterizes all estrogens regardless of their neuroprotective efficacy, an additional hydrogen bond interaction occurs between the atom ND1 of the residue His 524 and the hydroxyl group in the D ring of 17β-estradiol, serving as a main contributor to the intermolecular interaction that accounts for the high binding affinity of 17β-estradiol in comparison to the other 3 estrogens. A double bond in the B ring of the structures of equilin and Δ^8,9^-dehydroestrone but absent in estrone enhances the VDW interactions of estrogens with the vicinal residues situated in the binding site of the receptor. These analyses indicate that intermolecular hydrogen bond interactions are essential for estrogen binding to ER, and the steroidal skeletal structural change contributes to the differential binding affinities of estrogens as well. In addition, the neuroprotective efficacy of estrogens in primary basal forebrain neurons correlates to their interactions with ER. These data provide a computational chemistry platform upon which to predict the neuroprotective efficacy of estrogen molecules.

## Discussion

### The neuroprotective profile of select estrogens within CEE that are suggestive for clinical use

Taken together, the present study demonstrates that select estrogenic components within CEE are protective against neurodegenerative insults induced by β-amyloid_25–35 _and excitotoxic glutamate in primary basal forebrain neurons. Results of these analyses indicate that 3 of the 10 estrogens tested, 17β-estradiol, equilin and Δ^8,9^-dehydroestrone, were consistently neuroprotective against β-amyloid_25–35_-induced decline in membrane integrity and mitochondrial function. Further, our analyses indicate that coadministration of these neuroprotective estrogens can enhance the magnitude of their neuroprotective efficacy as evidenced by the observation that the above 3 estrogens, when used individually, were ineffective at the plasma dose whereas these same estrogens, when used in combination, were protective against excitotoxic glutamate-induced neuronal compromise of both the plasma membrane and mitochondrial function. The neuroprotective effects of combined estrogens could be synergistic in instances where a single estrogen exhibits no impact on neuronal viability, while the combined use of several estrogens induces a significant increase of neuronal viability. Another possibility is that since the concentrations of estrogens used in the present study were very low, consistent with the therapeutic plasma levels of these estrogens following a single oral dose of 0.625 mg CEE, the capacity of multiple estrogens to induce significant increase in markers of neuronal viability may simply be a threshold concentration issue. The low concentration of a single estrogen was not sufficient to increase neuronal defense mechanisms whereas a combination of multiple estrogens reached threshold concentration. In addition to the threshold concentration issue, the estrogen specificity requirement impacts estrogen efficacy as well, since not all estrogens were proved to be neuroprotective based on a quite sensitive measurement of LDH release.

Overall, the present study indicates that a combination of select neuroprotective estrogens at low concentrations could provide better therapeutic potential than administering a single estrogen. A formulation composed of both neuroprotective and non-protective estrogenic components may result in a compromised outcome through a competitive mechanism with multiple estrogens of varying efficacy competing for the same site of action with varying degrees of activation capability in the brain. Data from the present study suggest that, instead of using a complex formulation of estrogens with varying degrees of efficacy in the brain, using a formulation that is selectively efficacious for the brain could yield greater therapeutic benefit to promote neurological health and prevent neurodegeneration in postmenopausal women.

### Different biochemical markers based on different cellular processes may yield different responses

An important issue emerging from the present study relates to the biochemical markers used to assess the neuronal viability. For the LDH release assay, since the extracellular LDH remains stable for days, the magnitude of relative release of LDH correlates in linear fashion with the number of damaged neurons in the culture. The LDH release assay has been demonstrated to be a reliable indicator of cellular plasma membrane integrity and proved to be a consistent marker for estrogen efficacy in protecting neuronal membrane against neurotoxic insults in neurons [38]. The ATP and MTT assays are considered reliable indicators of cellular metabolic activity dependent upon mitochondrial functional integrity determined by the measurements of the cellular mitochondrial energy utilization and respiratory chain activity, respectively. In the present study, select estrogens that were neuroprotective against LDH release induced by neurotoxic β-amyloid_25–35 _or excitotoxic glutamate were ineffective in promoting neuronal metabolic integrity, indicating that neuronal membrane damage may be more easily protected and repaired than damage to neuronal mitochondrial function. The combined use of these select estrogens led to an enhanced neuroprotective efficacy, indicating that the neuroprotective efficacy exhibited by multiple estrogens could reach the threshold concentration sufficient for the maintenance of neuronal mitochondrial function. These analyses implicate a molecule that can protect neurons against plasma membrane damage reflected by the measurement of the LDH level in the culture medium does not necessarily have the same potential to maintain or reduce the damage to the cellular mitochondrial function. Further, these data suggest that one cannot expect these biochemical neuroprotective indexes to have equivalent responses to a chemical molecule because they are biochemical markers of distinct cellular processes. Therefore, investigations using multiple biochemical neuroprotective markers based on different cellular processes are necessary in order to determine the overall neuroprotective potential of an estrogenic molecule against the toxic insults in neurons.

In addition, it should be noted that the profile of neuroprotective efficacy of test estrogens demonstrated in the present study is associated with the therapeutic plasma levels of these estrogens achieved following a single oral dose of 0.625 mg CEE. For example, although pretreatment with 10 pg/ml of 17β-estradiol was effective in protecting neurons against β-amyloid_25–35_-induced intracellular ATP decline, an indication of neuronal mitochondrial dysfunction, such protection was not significant in neurons exposed to excitotoxic glutamate. One possible explanation is that compromise of neuronal mitochondrial function resulting from a slowly degenerative process, such as β-amyloid exposure, allows for cellular neuroprotective adaptations following β-amyloid exposure, whereas a rapid neurodegenerative insult, such as excitotoxic glutamate exposure, does not allow for upregulation of neuronal defense mechanisms but instead relies solely on existing levels of defense mechanisms. Consistent with this hypothesis, both β-amyloid and excitotoxic glutamate-induced neurodegeneration are associated with calcium influx, however the temporal pattern and magnitude of the calcium rise are quite different. β-Amyloid exposure induces a slow and modest rise in intracellular calcium whereas excitotoxic glutamate induces a rapid and robust rise in intracellular calcium [39]. However, the calcium-mediated neuronal dysfunction in response to excitotoxic glutamate can be overcome with either the combined use of multiple estrogens, as demonstrated in the present study, or the administration of a higher concentration of a single estrogen, such as 17β-estradiol, as evidenced by our previous studies, in which pretreatment of neurons with 10 ng/ml of 17β-estradiol induced a sustained mitochondrial respiration in the presence of high calcium [40,41].

### Structure and neuroprotective activity predict modes of actions by estrogens

Analyses of the chemical structures of both neuroprotective and non-neuroprotective estrogens within CEE indicate that both types of estrogens contain a phenolic A ring. Thus, it raises a question with respect to the necessity of this structural moiety in allowing estrogenic neuroprotection in the brain. A representative classical nuclear ER-independent mechanism proposed by Simpkins and colleagues suggests that estrogenic neuroprotection can be conferred by a phenolic A ring in the steroid structures of estrogens, which serves as a free-radical scavenging moiety by donating a proton that saturates neurodegenerative agent-induced unpaired reactive oxygen species [42-44]. An example in support of this hypothesis is 17α-estradiol, which is 100-fold less potent than its optical isomer, 17β-estradiol, in inducing classical estrogenic actions including uterotrophic stimulation and MCF7 cell proliferation, is as potent and efficacious as 17β-estradiol in a number of neuroprotection assays [44]. Our present study provided further evidence for 17α-estradiol's neuroprotective potency. Further, Simpkins et al demonstrated that in the hippocampal HT-22 cell line, a number of estrogens synergistically interacted with glutathione to increase the neuroprotective potency of estrogens [45]. The interaction between glutathione and endogenous estrogen 17β-estradiol-induced neuroprotection was also found in SK-N-SH human neuroblastoma cells and primary cortical neurons [46]. In addition, Simpkins and colleagues indicated that 17β-estradiol-induced promotion of neuronal survival in SK-N-SH cells is associated with the increased activity of neuronal nitric oxide synthase [47]. Other potential mechanisms of actions including estrogen interactions with signal transduction pathways and induction of mitochondrion-localized anti-apoptotic proteins have also been associated with the neuroprotective effects of phenolic A ring estrogens [48,49].

An alternative to an ER-independent mechanism of estrogen-induced neuroprotective functions is an ER subtype-dependent mechanism. ERα and ERβ are expressed in the brain with differential abundance in different brain regions [50]. Multiple factors modulate the relative contribution of the two ER subtypes to the outcomes of neuroprotection including the type of model system, the brain region and the cell type under investigation, and even the administered dose of estrogens [51]. For instance, in an *in vivo *ischemia model, Dubal and colleagues demonstrated that ERα, but not ERβ, is required for the protective effects of 17β-estradiol against ischemia-induced injury [52]. In contrast, Sawada and Shimohama showed that ERβ mediates estrogen neuroprotection in mesencephalon dopaminergic neurons that exclusively express ERβ [53]. Recent studies from Dorsa and colleagues demonstrated that both ERα and ERβ are involved in mediating estrogen neuroprotection against β-amyloid-induced toxicity and oxidative stress in HT-22 cells that stably express either ERα or ERβ, and that this ER-mediated neuroprotection requires the activation of the MAP kinase pathway [54,55]. Studies from our laboratory using ER subtype-selective agonists further indicate a near equivalent contribution of ERα and ERβ to estrogen-induced neuroprotection and upregulation of the neuroprotective gene, Bcl-2, in primary hippocampal neurons [38].

We speculate that estrogen induction of neuroprotection can be mediated by either ER-independent or ER-dependent pathways depending upon the concentration of estrogen administered. An ER-independent anti-oxidant property is much likely the dominant factor contributing to estrogen neuroprotection at suprapharmacological doses (μM range). In contrast, at a relatively low therapeutically relevant concentration range (pM or nM range), an ER-dependent signalling pathway is likely to be the main mediator of estrogen induction of neuroprotection [56]. Our analysis revealed that estrone at 5 nM was ineffective in enhancing neuronal defense systems in response to neurotoxic insults. In analyses by Bae and colleagues, 10–100 μM estrone protected against oxidative stress-induced cell death in mouse cortical neurons, whereas, estrone at a concentration of less than 3 μM was ineffective. Together, these data indicate that estrone is an ineffective activator of the brain ER and the associated signalling events required for estrogen neuroprotection at a low therapeutically relevant concentration, whereas, at a suprapharmacological dose, estrone can function as an effective antioxidant.

Our investigation of the intermolecular interactions of estrogens with ERα as well as the correlation between the estrogen structures with their neuroprotective activities at low concentrations comparable to their plasma levels revealed that although 4 representative estrogen analogues, 17β-estradiol, equilin, Δ^8,9^-dehydroestrone and estrone, bind to ERα in a similar binding mode in both conformation and orientation, their structural small variances still result in slightly different intermolecular energies with ERα, which in turn could impact their neuroprotective efficacy as evidenced by the parallel coincidence between the intermolecular energies among estrogens and their neuroprotective activities. Furthermore, we found that the presence of a phenolic A ring did not predict which estrogens, at least at a relatively low therapeutic concentration, would be full neuroprotective estrogens as multiple estrogens represented by estrone which was not protective in most conditions but contains a phenolic A ring. This suggests that the presence of a phenolic A ring in estrogens may be essential, but not entirely sufficient for estrogen-inducible neuroprotection at both the cellular plasma membrane and mitochondrial function mediated by an ER-dependent signalling pathway. In comparison, the phenolic A ring may be the crucial structural feature for allowing induction of neuroprotection by a relatively high concentration of estrogen through its anti-oxidant property. Further, we hypothesize that estrogenic neuroprotection is irrespective of activation of classical nuclear ERs but may be mediated through a non-nuclear ER-mediated signalling pathway, as supported by a increasing number of studies for a membrane-localized ER in regulating estrogen functions in neurons [50].

## Conclusion

In conclusion, the present study demonstrates that select, not all, estrogens within the complex formulation of CEE at concentrations commensurate with their plasma levels achieved after an oral administration of 0.625 mg CEE in women, contribute to its neuroprotective efficacy. Coadministration of select neuroprotective estrogens can lead to a greater magnitude of neuroprotection of neuronal plasma membrane and mitochondrial function. These data provide important insights into the rational design and development of a composition of estrogen therapy to alleviate climacteric symptoms, promote neurological health and prevent age-related neurodegeneration, such as AD, in postmenopausal women. A more effective and safer *brain-selective *estrogen formulation than CEE could be developed through a thorough analysis of the neuroprotective efficacy of those estrogens of interest under physiological conditions.

## Methods

### Neuronal cultures

The use of animals was approved by the Institutional Animal Care and Use Committee (IACUC) at the University of Southern California. Primary cultures of basal forebrain neurons were obtained from embryonic day 18 (E18d) Sprague-Dawley rat fetuses as previously described [15,36]. Briefly, after dissection from the brains of the rat fetuses, the septal basal forebrain was treated with 0.02% trypsin in Hank's balanced saline solution (HBSS, 137 mM NaCl, 5.4 mM KCl, 0.4 mM KH_2_PO_4_, 0.34 mM Na_2_HPO_4_·7H_2_O, 10.0 mM glucose, and 10.0 mM HEPES) for 5 min at 37°C and dissociated by repeated passage through a series of fire-polished constricted Pasteur pipettes. Approximately 10^5 ^neurons/ml were plated onto poly-D-lysine (10 μg/ml)-coated 24-well and 96-well culture plates for biochemical analyses. Neurons were grown in a phenol red-free Neurobasal medium (NBM, Invitrogen) supplemented with B27, 5 U/ml penicillin, 5 μg/ml streptomycin, 0.5 mM glutamine, and 25 μM glutamate at 37°C in a humidified 10% CO_2 _atmosphere for the first 3 days and NBM without glutamate afterwards. Cultures grown in serum-free NBM yield approximately 99.5% neurons and 0.05% glia cells.

### Estrogen treatment

The estrogens were tested at concentrations commensurate with their plasma levels achieved following a single oral dose of 0.625 mg CEE, including estrone (5000 pg/ml), 17α-estradiol (10 pg/ml), 17β-estradiol (10 pg/ml), equilin (1000 pg/ml), 17α-dihydroequilin (1000 pg/ml), 17β-dihydroequilin (40 pg/ml), equilenin (300 pg/ml), 17α-dihydroequilenin (27 pg/ml), 17β-dihydroequilenin (27 pg/ml) and Δ^8,9^-dehydroestrone (300 pg/ml). All estrogens were purchased from Steraloids with the exception of 17α-dihydroequilenin, 17β-dihydroequilenin and Δ^8,9^-dehydroestrone, which were gifts from Dr. Michael Dey and Dr. Richard Lyttle of Wyeth-Ayerst Laboratories. The chemical structures of these estrogens are listed in Fig. 1. A vehicle alone-treated control group and a neurotoxin alone (β-amyloid_25–35 _or glutamate)-treated group were included in all experiments.

### β-Amyloid_25–35 _exposure

β-Amyloid_25–35 _purchased from Bachem was dissolved in sterile distilled water at a concentration of 1 mg/ml as a stock solution. This stock was aliquoted and stored at -20°C. Three-day-old basal forebrain neurons were pretreated with test estrogens for 4 days followed by exposure to freshly prepared 8 μg/ml β-amyloid_25–35 _in NBM in the presence of test estrogens for 24 hr at 37°C. Following this exposure, the culture medium was replaced with fresh β-amyloid_25–35_-free NBM containing test estrogens. Cultures were returned to the culture incubator and allowed to incubate for additional 24 hr prior to cell viability measurements on the following day.

### Glutamate exposure

Three-day-old basal forebrain neurons were pretreated with test estrogens for 4 days followed by exposure to 200 μM glutamate for 5 min at room temperature in HEPES-buffered saline solution (HBS) containing 100 mM NaCl, 2.0 mM KCl, 2.5 mM CaCl_2_, 1.0 mM MgSO_4_, 1.0 mM NaH_2_PO_4_, 4.2 mM NaHCO_3_, 10.0 mM glucose and 12.5 mM HEPES. Immediately following glutamate exposure, cultures were washed once with HBS and replaced with fresh NBM containing test estrogens. Cultures were returned to the culture incubator and allowed to incubate for an additional 24 hr prior to cell viability measurements on the following day.

### LDH assay

Lactate dehydrogenase (LDH) is a stable cytoplasmic enzyme present in all cells including neurons. It is rapidly released into the cell culture supernatant when the cell plasma membrane is damaged. Thus, the LDH level in the culture medium is a reliable biochemical index for neuronal plasma membrane damage. In this study, LDH release from the cytosol of damaged neurons into the culture medium following β-amyloid_25–35 _or glutamate exposure was measured using a Cytotoxicity Detection Assay (Boehringer Mannheim Biochemicals), which determines the LDH activity in the culture medium to enzymatically convert the lactate and NAD^+ ^to pyruvate and NADH. The tetrazolium salt produced in the enzymatic reaction was then reduced to red formazan in the presence of H^+^, thereby allowing a colormetric detection for neuronal membrane integrity.

### ATP assay

Intracellular ATP (adenosine triphosphate) levels were determined by a luciferin/luciferase-based method with a Bioluminescent Assay (Sigma-Aldrich), which uses ATP, a required co-factor of the luciferase reaction, producing oxyluciferin and releasing energy in the form of luminescence that is proportional to the amount of ATP present, which further signals the presence of metabolically active cells. After the culture medium supernatant was taken for the LDH assay and the rest of the medium was aspirated off, the ATP-releasing reagent was added to the culture plate to solubilize cellular membrane which allowed the release of intracellular ATP into the medium. The generated luminescence through the luciferase reaction was quantified with a TD-20e TURNER luminometer.

### MTT assay

Neuronal metabolic viability was further determined using a MTT (3-(4,5-dimethylthiazol-2-yl)-2,5-diphenyltetrazolium bromide, Boehringer Mannheim Biochemicals) assay, which measures the enzymatic activity of the cellular mitochondrial respiratory chain through the conversion of yellow MTT to a purple formazan crystal by metabolically active cells. MTT was added to the culture plate at a final concentration of 0.5 mg/ml and incubated at 37°C for 4 hr. The reaction was stopped by the addition of 1 mM HCl in 10% SDS. Formazan crystals were dissolved overnight and absorption was measured with a spectrophotometer at 575 nm.

### Molecular modelling

All the calculations were performed on a SGI Octane graphical workstation equipped with the IRIX 6.5 operating system (Silicon Graphics Inc.) using the molecular modeling software package InsightII 2000 (Accelrys Inc.). The 3D structures of estrogens were constructed and energy minimized via both molecular mechanics and dynamics simulation. The binding modes (position and orientation) of estrogens in the ligand binding site of ER were predefined by superimposing the optimized 3D structures of estrogens and the crystallographic structure of 17β-estradiol bound with its cognate receptor [58]. Then the structure of 17β-estradiol was removed from the complex and the remaining objects were associated as a new assembly. Following the hydrogenation of the system based on a target pH of 7.0, the resultant complex, which served as the initial binding model of estrogens in complex with ER, was energetically optimized by the Discover calculation under a consistent-valence forcefield (CVFF). The initial estrogen-ER complex system was first energetically minimized with the receptor atoms fixed by consecutively executing 800 steps of steepest descents (SD) minimization and then 500 steps of conjugate gradients (CG) minimization. The refined complex system was then relaxed by removing the constraint on the residues within 7 Å of estrogens and further energetically optimized by applying 800 steps of SD and 500 steps of CG, followed by a 20 ps molecular dynamics simulation at 300 K. Conformations were written to a history file every 1 ps and the collected conformations underwent further molecular mechanics minimization by performing 800 steps of SD and 500 steps of CG. The yielded estrogen bound conformation with the lowest energy was chosen as the final binding solution.

### Data analyses

Results from LDH, ATP and MTT assay are normalized against the data obtained from neurotoxin alone-treated cultures. Data are presented as the mean ± SEM derived from at lease three separate experiments. Statistically significant differences between groups were determined by a one-way analysis of variance (ANOVA) followed by a Newman-Keuls *post hoc *analysis.

## Competing interests statement

The author(s) declare that they have no competing interests.

## Authors' contributions

LZ carried out the analyses presented in the manuscript and drafted the manuscript. RDB conceived of the study, participated in its design and finalized the manuscript. All authors read and approved the final manuscript.

## Figures and Tables

**Table 1:** Efficacy of select estrogens within CEE to reduce LDH release following exposure to β-amyloid_25–35 _in basal forebrain neurons. Neuronal cultures were pretreated with indicated estrogens for 4 days followed by a 24 hr exposure to 8 μg/ml β-amyloid_25–35_. The culture medium was replaced and cultures were allowed to incubate for an additional 24 hr followed by determination of LDH release into the medium. Data are represented as mean ± SEM (n = 7 – 24 from 3 separate experiments). ** *P *< 0.01 and *** *P *< 0.001 compared to β-amyloid_25–35 _alone-treated cultures.

Treatment (pg/ml)	LDH Release (% of β-amyloid_25–35 _alone group)
Control	36.11 ± 1.60***
β-amyloid_25–35 _alone	100.00 ± 2.26
17α-estradiol (10)	86.62 ± 2.99**
17β-estradiol (10)	84.20 ± 1.47***
Equilin (1000)	89.01 ± 1.74**
Equilenin (300)	81.49 ± 3.06***
17α-dihydroequilin (1000)	89.96 ± 3.88**
17β-dihydroequilin (40)	101.53 ± 2.00
Estrone (5000)	99.33 ± 4.43
Δ^8,9^-dehydroestrone (300)	85.82 ± 2.07***

**Table 2:** Efficacy of select estrogens within CEE to sustain the intracellular ATP level following exposure to β-amyloid_25–35 _in basal forebrain neurons. Neuronal cultures were pretreated with indicated estrogens for 4 days followed by a 24 hr exposure to 8 μg/ml β-amyloid_25–35_. The culture medium was replaced and cultures were allowed to incubate for an additional 24 hr followed by determination of the intracellular ATP level. Data are represented as mean ± SEM (n = 7 – 24 from 3 separate experiments). ** *P *< 0.01 and *** *P *< 0.001 compared to β-amyloid_25–35 _alone-treated cultures.

Treatment (pg/ml)	ATP Level (% of β-amyloid_25–35 _alone group)
Control	203.37 ± 11.67***
β-amyloid_25–35 _alone	100.00 ± 2.07
17α-estradiol (10)	103.83 ± 3.76
17β-estradiol (10)	131.32 ± 4.56**
Equilin (1000)	114.58 ± 3.56
Equilenin (300)	96.44 ± 4.07
17α-dihydroequilin (1000)	110.00 ± 2.75
17β-dihydroequilin (40)	110.75 ± 2.46
Estrone (5000)	128.84 ± 4.14**
Δ^8,9^-dehydroestrone (300)	125.05 ± 1.42**

**Table 3:** Efficacy of select estrogens within CEE to reduce LDH release following exposure to excitotoxic glutamate in basal forebrain neurons. Neuronal cultures were pretreated with indicated estrogens for 4 days followed by a 5 min exposure to 200 μM glutamate. The culture medium was replaced and cultures were allowed to incubate for an additional 24 hr followed by determination of LDH release into the medium. Data are represented as mean ± SEM (n = 11 – 14 from 3 experiments). ** *P *< 0.01 and *** *P *< 0.001 compared to glutamate alone-treated cultures.

Treatment (pg/ml)	LDH Release (% of glutamate alone group)
Control	47.49 ± 2.69***
Glutamate alone	100.00 ± 2.89
17β-estradiol (10)	57.55 ± 3.00**
Equilin (1000)	64.02 ± 3.03**
Estrone (5000)	85.46 ± 3.22
Δ^8,9^-dehydroestrone (300)	66.05 ± 2.74**

**Table 4:** Efficacy of select estrogens within CEE to sustain the intracellular ATP level following exposure to excitotoxic glutamate in basal forebrain neurons. Neuronal cultures were pretreated with indicated estrogens for 4 days followed by a 5 min exposure to 200 μM glutamate. The culture medium was replaced and cultures were allowed to incubate for an additional 24 hr followed by determination of the intracellular ATP level. Data are represented as mean ± SEM (n = 11 – 14 from 3 experiments). ** *P *< 0.01 compared to glutamate alone-treated cultures.

Treatment (pg/ml)	ATP Level (% of glutamate alone group)
Control	128.72 ± 4.21**
Glutamate alone	100.00 ± 1.50
17β-estradiol (10)	109.38 ± 2.91
Equilin (1000)	108.44 ± 1.86
Estrone (5000)	93.41 ± 3.85
Δ^8,9^-dehydroestrone (300)	111.80 ± 4.51

**Table 5:** Efficacy of select estrogens within CEE to reduce LDH release and sustain MTT reduction following exposure to excitotoxic glutamate in basal forebrain neurons. Neuronal cultures were pretreated with indicated estrogens for 4 days followed by a 5 min exposure to 200 μM glutamate. The culture medium was replaced and cultures were allowed to incubate for an additional 24 hr followed by determination of LDH release into the medium and MTT reduction. Data are represented as mean ± SEM (n = 17 – 33 from 3 experiments). * *P *< 0.05, ** *P *< 0.01 and *** *P *< 0.001 compared to glutamate alone-treated cultures.

Treatment (pg/ml)	LDH Release (% of glutamate alone group)	MTT Reduction (% of glutamate alone group)
Control	41.59 ± 2.85***	194.49 ± 3.52***
Glutamate alone	100.00 ± 1.25	100.00 ± 1.51
17α-estradiol (10)	83.03 ± 1.91**	98.06 ± 1.39
17β-estradiol (10)	92.29 ± 1.91**	98.81 ± 1.93
Equilin (1000)	86.37 ± 2.19**	105.76 ± 3.36
Equilenin (300)	88.00 ± 2.55**	103.62 ± 1.86
17α-dihydroequilin (1000)	90.68 ± 2.21**	102.22 ± 2.25
17α-dihydroequilenin (27)	84.74 ± 3.02**	91.10 ± 5.63
17β-dihydroequilenin (27)	90.87 ± 2.99*	113.17 ± 3.65
Δ^8,9^-dehydroestrone (300)	86.69 ± 2.20**	102.88 ± 2.71

**Table 6:** Intermolecular interaction between estrogens and ERα. ^a^van der Waals energy; ^b^electrostatic energy.

Estrogens	Intermolecular Energy			H-Bond Distance	
	
	VDW^a^	Elect^b^	Total	d_1 _(Glu353:OE2)	d_2 _(His524:ND1)
17β-estradiol	-55.06	-20.43	-75.48	1.56	2.02
Equilin	-58.17	-12.33	-70.49	1.71	
Δ^8,9^-dehydroestrone	-58.40	-13.09	-71.49	1.67	
Estrone	-55.92	-12.76	-68.68	1.66	
